# Perceived stress among Brazilian anesthesiologists before and after a mindfulness-based program: preliminary findings

**DOI:** 10.1016/j.bjane.2025.844638

**Published:** 2025-05-12

**Authors:** Elisa Duarte Candido, DurvalCampos Kraychete

**Affiliations:** a Rede Mater Dei de Saúde, Salvador, BA, Brazil; b Universidade Federal da Bahia, Salvador, BA, Brazil

**Keywords:** Anesthesiologist, Mindfulness, Stress

Dear Editor,

Anesthesiologists are at increased risk of experiencing stress due to the high-responsibility environments in which they work within hospitals.^1^ They often lack autonomy and flexibility in managing their work schedules, typically adhere to long weekly working hours, and are required to maintain sustained attention and concentration during anesthetic procedures. In addition, they bear significant responsibility for patient’s clinical outcomes, must engage in complex interpersonal interactions with fellow anesthesiologists and surgeons, and frequently face fear of litigation as well as mounting pressure for productivity within surgical centers.^1^

Modifying the structural and organizational context of operating rooms and the daily routines of anesthesiologists is often more complex than equipping these professionals with strategies to respond to adversity in a less reactive, more observant and composed manner.^2^ Mindfulness-based intervention programs are accessible and well-established tools that have demonstrated potential to positively impact mental health and overall well-being, enhance workplace performance, reduce the incidence of medical errors and improve patient satisfaction.^3^ The aim of this study is to evaluate the impact of an eight-week Mindfulness program on perceived stress among Brazilian anesthesiologists using the PSS-10 questionnaire administered anonymously before and after the intervention.

This is a *quasi*-experimental pre-post study design involving data collection from a single group at two distinct time points: before and after the intervention. The sample size was calculated based on a paired Student’s *t*-test assuming an expected effect size of 0.80, a significance level (α) of 0.05 (5 %), and a corresponding confidence level of 95 % (1 – α), the estimated total sample size was 23 participants. The study participants were drawn from the population of 89 anesthesiologists affiliated with *Grupo Sam*, the anesthesiology team of *Rede Mater Dei* in Brazil, and exclusion criterion was regular Mindfulness practice or prior program completion.

Of the sampled population, 35 volunteers completed the initial PSS-10 questionnaire. The Mindfulness intervention was conducted between April and July 2023 in a synchronous online format, consisting of weekly 2-hour sessions over the course of eight weeks, and was delivered by NUMI (*Núcleo de Mindfulness*). Among the volunteers, 25 individuals completed both the pre and post intervention assessments. Of these, 16 participants completed the course, while 9 did not. Completion of the course is defined as attending at least 5 out of the 8 sessions with the first session being mandatory.

Data were analyzed using IBM SPSS Statistics version 26.0. Descriptive statistics were used for quantitative variables, and absolute (n) and relative (%) frequencies were used to describe categorical variables. To compare two measurements obtained from the same experimental unit at different time points a paired Student’s *t-*test was applied. Effect size was assessed using Cohen’s d coefficient, and results were considered statistically significant when the p-value was less than 0.05, corresponding to a 95% confidence level in the conclusions drawn.

Among participants who did not complete the course but responded to the questionnaire at both time points (*n* = 9), no statistically significant differences were observed in Perceived Stress Scale (PSS-10) scores between the pre and post intervention assessments (*p* = 0.773). The mean PSS score changed from 23.4 ± 7.1 (95% CI: 18.01 - 28.88) at baseline to 22.9 ± 3.7 (95% CI: 20.08 - 25.7) after the course, with a small effect size (*d* = 0.10). The median also remained stable, shifting from 20.0 (Q1–Q3: 17.5–32.0) to 22.0 (Q1–Q3: 20.0–26.5), suggesting no relevant change in perceived stress levels within this subgroup.

Among participants who completed the course (*n* = 16), a significant reduction in Perceived Stress Scale (PSS-10) scores was observed between the pre and post intervention assessments, with a statistically significant difference (*p* < 0.001; *t*(15) = 3.924) and a large effect size (*d* = 0.98). The mean score decreased from 21.4 ± 5.4 (95% IC: 18.55 - 24.32) before the course to 16.2 ± 4.7 (95% IC: 13.7 - 18.67) after the intervention. The median score also declined, from 22.0 (interquartile range: 17.0–25.0) at baseline to 16.5 (interquartile range: 13.0–20.3) post-intervention. Minimum and maximum values ranged from 12.0 to 32.0 before the course, and from 8.0 to 24.0 after the course, indicating not only a reduction in mean perceived stress levels but also a decrease in score dispersion following full participation in the intervention.

When comparing Perceived Stress Scale (PSS-10) scores between participants who completed the course (*n* = 16) and those who did not (*n* = 9), the groups showed similar results at the pre-intervention time point. However, a statistically significant reduction in PSS-10 scores was observed in the post-intervention assessment among those who completed the course.

At the pre-intervention time point, the mean PSS-10 scores were 21.4 ± 5.4 for the course completion group and 23.4 ± 7.1 for the non-completion group. The difference between the groups was not statistically significant (*p* = 0.434; *t*(23) = 0.797) with a small effect size (*d* = 0.34), suggesting baseline equivalence between the groups prior to the intervention.

At the post-intervention time point, a significant difference between the groups was observed: the mean score for the course completion group was 16.2 ± 4.7, while the non-completion group had a mean score of 22.9 ± 3.7. This difference was statistically significant (*p* = 0.001; *t*(23) = 3.705) with a large effect size (*d* = 1.60), indicating a substantial improvement in perceived stress levels among those who completed the course.

This study demonstrated that although both subgroups exhibited similar levels of perceived stress prior to the intervention (*p* = 0.434; *d* = 0.34), only the subgroup that completed the Mindfulness course showed a statistically significant reduction in PSS-10 scores at the end of the intervention (16.2 ± 4.7), compared to the non-completion subgroup (22.9 ± 3.7). This difference was statistically significant (*p* = 0.001) with a large effect size (*d* = 1.60) (Table 1).

**Table 1:** Comparative analysis of PSS scores between participants who completed and those who did not complete the course.

	Course	
PSS	Completed (*n* = 16)	Not completed (*n* = 9)
**Pre-course**		
Mean ± SD	21.4 ± 5.4	23.4 ± 7.1
Median (Q_1_ – Q_3_)	22.0 (17.0 – 25.0)	20.0 (17.5 – 32.0)
Minimum – Maximum	12.0 – 32.0	17.0 – 34.0
**Conclusion:**	*p* = 0.434; *t*_23_ = 0.797; *d* = 0.34	
	Completed = Not completed; Effect size: Small	
**Post-course**		
Mean ± SD	16.2 ± 4.7	22.9 ± 3.7
Median (Q_1_ – Q_3_)	16.5 (13.0 – 20.3)	22.0 (20.0 – 26.5)
Minimum – Maximum	8.0 – 24.0	18.0 – 29.0
**Conclusion:**	*p* = 0.001; *t*_23_ = 3.705; *d* = 1.60	
	Completed < Not completed; Effect size: Large	

Dataset: 25 participants.

Note: *p, p*-value from the independent samples Student’s *t*-test; *d*, Effect size (Cohen’s *d*).

These results indicate that completing the Mindfulness course may be associated with a significant reduction in perceived stress, reinforcing the effectiveness of the intervention in this population of anesthesiologists.

The final sample size was small (*n* = 16), which implies wider confidence intervals and reduced statistical power to detect differences. Nevertheless, a statistically significant difference was observed (*p* < 0.001). To address limitations related to statistical power, the effect size was calculated and found to be large (*d* = 0.98). This suggests that the observed difference in PSS-10 scores before and after the course is clinically relevant and unlikely to be due to chance.

The *quasi*-experimental design of this study inherently presents limitations, including the potential for selection bias and regression to the mean. Without a control group or random allocation, it is difficult to establish causal inference, as the observed effects may be confounded by external variables such as seasonal fluctuations in workload or other unmeasured factors. Incorporating a control group would strengthen internal validity and allow for a more accurate estimation of the effects directly attributable to the Mindfulness intervention.

It is noteworthy that, in the present study, 45.71 % of the sampled population completed the course, while 54.29 % did not, and 28.6 % of all enrolled participants did not attend any session. Given the documented benefits of Mindfulness-based programs, it is important to identify factors that may enhance engagement and participation among healthcare teams in this type of practice.

Data on the daily meditation practice volume for each participant were not collected, therefore, it was not possible to adjust the results for the covariate “hours of meditation.” Regular practice frequency is one of the core components of Mindfulness.^3^

Barriers such as workload related fatigue, personal life demands, and the course schedule, limit the feasibility of Mindfulness practice at both individual and institutional levels. Shorter interventions incorporating core components of Mindfulness may offer a viable solution for implementing the practice in the context of healthcare professionals.^4^

Additional strategies may include institutional support for conducting interventions during working hours; the creation of dedicated spaces within the workplace that encourage regular Mindfulness practice; flexible scheduling of course sessions; availability of asynchronous online courses; promotion of mobile meditation app usage; and ongoing education on the importance of mental health and the individual and institutional impact of stress on healthcare professionals.

Moreover, there is a lack of studies evaluating the long-term impact of such interventions on this professional population.^5^

The main contribution of the present study is the application of Mindfulness as an accessible tool with the potential to transform anesthesiologist’s relationship with their work environment without necessarily altering the inherent characteristics or operational logic of the surgical setting.

By bringing the discussion on the impact of Mindfulness in anesthesiologists into the scientific community, this study aims to contribute to professional self-care initiatives and raise awareness among institutions responsible for the specialty regarding the relevance and feasibility of implementing Mindfulness techniques in this context.

## Declaration of competing interest

The authors declare no conflicts of interest.
